# Multidimensional Aspects of* de qi* Sensations in MASS and ASQ Assessment: A Pilot Study

**DOI:** 10.1155/2017/6249329

**Published:** 2017-02-07

**Authors:** Jong-Chan Jang, Jaegyun Jung, Haebeom Lee, Young-Bae Park, Hyunho Kim

**Affiliations:** ^1^College of Korean Medicine, Kyung Hee University, Seoul 02447, Republic of Korea; ^2^College of Korean Medicine, Dongshin University, Naju 58245, Republic of Korea; ^3^Department of Human Informatics of Korean Medicine, Graduate School, Kyung Hee University, Seoul 02447, Republic of Korea; ^4^Department of Biofunctional Medicine and Diagnostics, College of Korean Medicine, Kyung Hee University, Seoul 02447, Republic of Korea

## Abstract

*Background. De qi* comprises varied senses depending on the individual. No single method can yet fully measure the multiple dimensions of* de qi* adequately.* Objective.* We examined the advantages of implementing multiple questionnaires for* de qi* measurement.* Methods.* Fourteen participants completed a preacupuncture questionnaire regarding their perception toward acupuncture treatment. After acupuncture stimulation at the HT7 point,* de qi* sensations were measured by MASS and ASQ. In groups with different levels of expectation, we compared the subtotal scores of each phase in the ASQ, as well as the VAS* de qi* intensity and MASS index using Kruskal-Wallis test. For the structural comparison of questionnaires, we first performed Spearman's rank correlation test between the scores of individual descriptors in MASS and ASQ. The subtotal scores of each phase in ASQ was compared with VAS* de qi* intensity and MASS index.* Results.* The subtotal score of the manipulation phase in ASQ strongly correlated with the VAS score of* de qi* intensity (Spearman's *ρ* = 0.654, *p* = 0.011) and MASS index (Spearman's *ρ* = 0.488, *p* = 0.076). MASS and ASQ showed strong correlations in certain analogous descriptors. Unpleasant perceptions toward acupuncture treatment did not significantly correlate with overall* de qi* intensity.* Conclusions. De qi* sensations in acupuncture treatment have multidimensional aspects. Intensity of stimulation, ASQ, and MASS index assess somewhat restricted aspects of* de qi*. Those questionnaires have exclusive differences of sets in spite of their strong intersections. Use of multiple questionnaires may enable a more comprehensive understanding of* de qi* properties and the elicitation of relevant construction in* de qi* features of acupuncture.

## 1. Introduction

Acupuncture involves sensory stimulation, which consequently evokes varied sensations upon insertion and manipulation of the needle [1, 2].* De qi* is a prerequisite term in Traditional Chinese Medicine (TCM) used to describe such composite sensations detected by the subject, practitioner, or both [3]. In clinical practice, it has historically been thought to be an indicator of appropriate acupuncture treatment and thus relates to therapeutic effectiveness. As* Neijing* states, the arrival of* qi* is “essential for acupuncture to be successful” [4]. A recent survey conducted by Yuan et al. reported that practitioners and patients both empirically expect greater efficacy in stronger* de qi* sensations [5]. Most importantly, a correlative link between needling sensation and the therapeutic efficacy of acupuncture has been suggested from studies by Choi et al. and Xiong et al. [6, 7]. Contradictory data do exist, leaving the matter to debate [8, 9]. Such attempts to associate* de qi* and the therapeutic effectiveness of acupuncture have since led investigators to measure the qualitative and quantitative aspects of* de qi*, leading to the development of the diverse scales currently used.

The earliest documented perceptions of* de qi* include* suan* (aching or soreness),* ma* (numbness or tingling),* zhong* (heaviness), and* zhang* (fullness or distention) [3]. In 1989, Vincent et al. first identified the seven components of needle sensation via a component analysis of data obtained from 125 subjects, using the McGill Pain Questionnaire (MPQ) [10]. MacPherson and Asghar later pointed out that* de qi* sensations, which were largely consistent with the demonstrations of Vincent et al., were distinguishable from acute pain at the needling site [11]. In view of this report, Kong et al. suggested a scale entitled the “Subjective Acupuncture Sensation Scale (SASS),” characterized by a substitution of the MPQ in rating the sensations of* de qi*. SASS consisted of nine predefined descriptors and one subjective component specified by the subjects in their own words. Kong and colleagues later modified the scale for a more comprehensive embracement of sensations and termed it “Massachusetts General Hospital Acupuncture Sensation Scale” (MASS). MASS includes 12 descriptors, incorporating additional sensations such as “warmth” or “coolness” [12]. In 2008, Kim et al. reported a novel scale entitled “Acupuncture Sensation Questionnaire” (ASQ), which detected stepwise sensations in each phase of the acupuncture treatment: insertion, manipulation, and retention [13].

This study aimed to compare two of the validated questionnaires for assessing* de qi* sensations, namely, MASS and ASQ.* De qi* is a multidimensional concept that encompasses varied sensations with distinct intensities, at different phases of acupuncture treatment. Yet, there is no single widely used questionnaire to fully assess such responses with comprehensiveness. While some descriptors overlap with each other, exclusive realms remain for most of the scales being used. We evaluated the relevance of uniform or analogous descriptors of MASS and ASQ to demonstrate concurrency. To determine the specific phase of treatment that mainly affects the intensity of* de qi* in subjects, a correlational study between the scores of* de qi* intensity in MASS and the respective subtotal points of each phase in ASQ was performed. Also, an additional link between the intensity of* de qi* measured in MASS and the individual descriptors presented in ASQ was studied to delineate the dominant constituents of* de qi*. Finally, we examined the effects of unpleasant perceptions toward acupuncture on the scores of both questionnaires, as* de qi* has been reported to be associated with psychological factors such as fear or expectation following prior experience [14, 15].

## 2. Materials and Methods

### 2.1. Subjects

The clinical trial was performed in Kyung Hee University Korean Medicine Hospital; 15 healthy participants were recruited through in-person or telephone interviews. Subjects aged 20 to 49 years that were able to communicate with researchers by following verbal orders and completing questionnaires were included. Exclusion criteria were as follows: (1) abnormal cardiac cycles, (2)* qi-gong* practitioners and athletes, (3) history of hypertension, arrhythmia, ischemic heart disease, or other cardiovascular diseases, and (4) history of cardiovascular diseases that required drug treatment within one month of the trial. Among 15 volunteers, one was excluded according to the exclusion criteria. Therefore, a total of 14 participants (four males and ten females) were included in this study. They were aged between 23 and 33; the mean age was 27.07 with a standard deviation of 2.67 (years). The clinical trial protocol was approved by the Institutional Review Board of Kyung Hee University Korean Medicine Hospital (number KOMCIRB-150420-HR-015).

### 2.2. Questionnaires

#### 2.2.1. Acupuncture Perception Questionnaire

In order to assess the correlation between psychological factors and the MASS/ASQ scores, we used a self-developed questionnaire about the subjects' perception regarding acupuncture treatment. The two items included in the questionnaire were as follows: (1) “How many times have you experienced medical acupuncture treatment(s) until now?” (2) “If you have experienced the following feelings (fear, discomfort, pain) during medical acupuncture treatment(s), please mark the category that best applies to your experience.” For each feeling, patients were given a choice from “none,” “moderate,” “severe,” and “very severe.”

#### 2.2.2. Visual Analog Scale (VAS)

The visual analog scale (VAS) is an objective, easy, and reliable way to assess the intensity of the needle sensation of* de qi* [16]. After 5 minutes of rest following the needle removal, researchers asked each participant to use the VAS to quantify their overall intensity of* de qi* sensation. There was one item (intensity of* de qi*) presented on 0 (no needle sensation) to 10 (extremely strong needle sensation) continuous bar, and the subject was asked to mark the point that best applies to their experience.

#### 2.2.3. Massachusetts General Hospital Acupuncture Sensation Scale (MASS)

MASS, a questionnaire comprised of 12 descriptors, is a revised version of SASS, which was previously used in healthy subjects. MASS also includes one supplementary scale so that participants may express their* de qi* sensation in their own words [17]. Each of the 12 descriptors was presented in the form of VAS. The MASS index, a single value that could represent the full multivariate breadth and depth of acupuncture sensations, was calculated using previously published methods [12]. In brief, the weighted average of ratings of each descriptor was calculated using exponential smoothing.

#### 2.2.4. Acupuncture Sensation Questionnaire (ASQ)

ASQ is another way to assess* de qi* sensation. ASQ, as opposed to MASS, does not apply the conventional method of a single time-independent assessment; instead, it divides the whole procedure of acupuncture treatment into three phases; needle insertion (Phase 1), manual stimulation (Phase 2), and needle retention (Phase 3). ASQ includes 19 items: 3 for needle insertion, 9 for manual stimulation, and 7 for needle retention. Each participant was asked to rate their* de qi* sensation on VAS of these 19 items.

### 2.3. Clinical Trial Procedure

**Figure 1:**
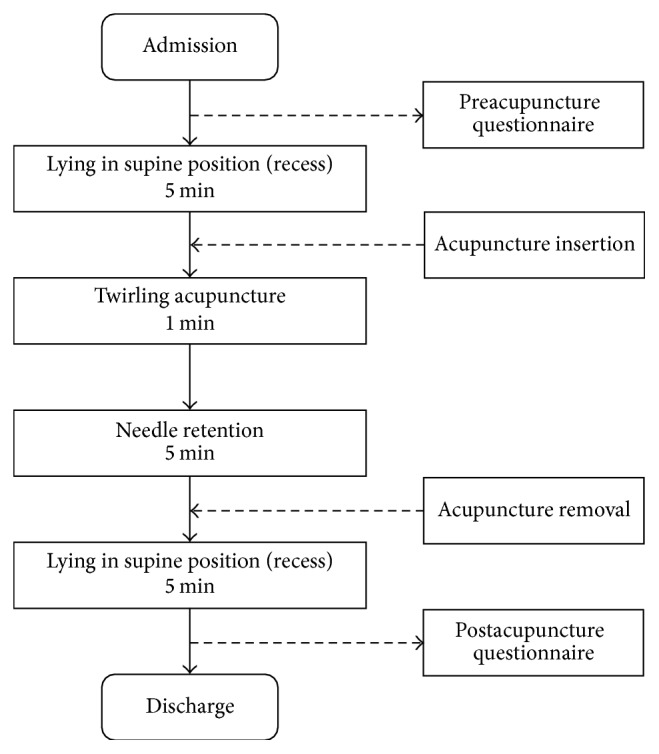
Flow diagram of the study.

After the provision of written information about the trial, all subjects signed the informed consent form. First, each participant completed the questionnaire about their perception toward acupuncture treatment and rested on a bed for 5 minutes in the supine position. Prior to treatment, the practitioner sterilized the skin of each participant with a 75% alcohol application. Acupuncture treatment was performed on HT7 of each participant's dominant hand located on the anteromedial aspect of the wrist, radial to the flexor carpi ulnaris tendon, on the palmar crease [18]. With the aid of a guided tube, sterile needles with a diameter of 0.30 mm and a length of 40 mm (Dongbang Acupuncture, Kyunggi-do, Korea) penetrated the skin upright at approximately 5 to 10 mm. Immediately after insertion, the guided tube was removed. The needle was then rotated 120 times per minute at angles of ±180°, in order to generate the needle sensation known as* de qi*. After 1 minute of manipulation, the needle was retained for 5 minutes and then removed. All acupuncture treatments were performed by a Korean Medicine Doctor who had 2 years of clinical experience. Participants rested for 5 minutes on a bed in the supine position after the treatment and were asked to complete two postacupuncture questionnaires: MASS and ASQ. The overall procedure is presented in Figure 1.

### 2.4. Data Analysis

Statistical analysis and data visualization were performed with the SPSS 18.0 Software (SPSS Inc., Chicago, IL, USA) and R 3.2.3. (R Core Team, Vienna, Austria). Kruskal-Wallis test was used in order to compare the ASQ scores of each phase (1–3), overall intensity of de qi, and MASS index in groups with different perception. Correlation analysis between (1) MASS descriptors and ASQ items and (2) subtotal scores of each phase in ASQ and VAS score of* de qi *intensity/MASS index was performed with Spearman's rank correlation test. Significance of gender differences in outcomes was tested using Fisher's exact test and Mann–Whitney *U* test. The *p* values of less than 0.05 were considered to be statistically significant. All confidence intervals were reported at the 95% confidence level.

## 3. Results

### 3.1. Outcomes: Acupuncture Perception Questionnaire, ASQ, VAS, MASS, and the MASS Index

All participants previously experienced acupuncture treatment more than 10 times. Results of the perception questionnaire are shown in Table 1. In detail, 3 out of 14 participants chose “none”; 9 chose “moderate”; and 2 chose “severe” for the category “fear.” For the category “discomfort,” “none” and “moderate” were selected by 6 participants each, while “severe” was selected by 2 participants. There were 8 participants who marked “moderate” for the category “pain,” and 3 participants selected “none” and “severe,” respectively. None of the participants expressed “very severe” for all 3 items.  Table 2 shows the mean scores and 95% confidence interval for ASQ. Results of the MASS descriptors and MASS index are presented as a mean score and 95% confidence interval in Table 3. Table 3 also includes the average score of* de qi* intensity that each participant experienced, which was acquired using VAS.

### 3.2. Correlational Analysis between ASQ, MASS, and VAS

**Figure 2:**
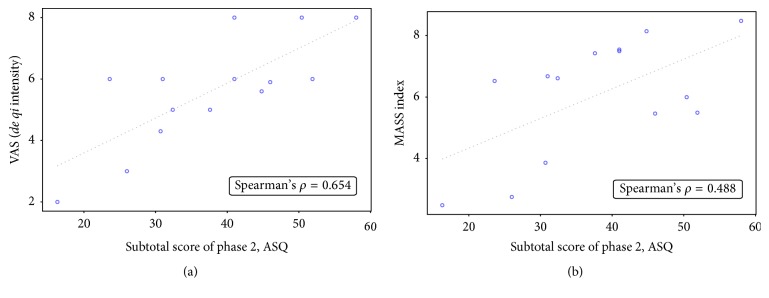
Correlational analysis between subtotal score of phase 2 in Acupuncture Sensation Questionnaire (ASQ) and (a) visual analog scale (VAS) score of* de qi* intensity and (b) Massachusetts General Hospital Acupuncture Sensation Scale (MASS) index (*n* = 14). The subtotal score of phase 2 in ASQ had a positive correlation with both VAS score of* de qi *intensity (Spearman's *ρ* = 0.654, 95% CI = 0.190 to 0.880) and MASS index (Spearman's *ρ* = 0.488, 95% CI = −0.0569 to 0.809).

**Figure 3:**
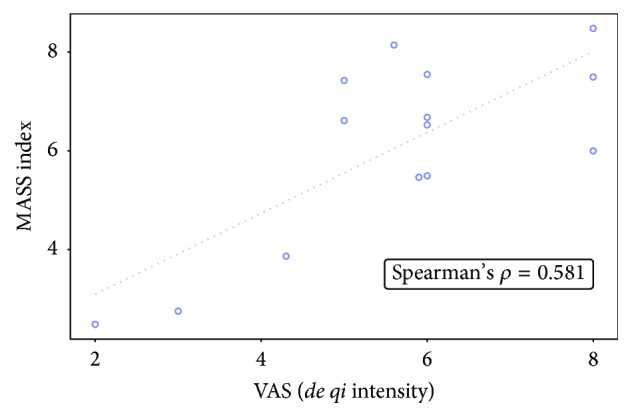
Correlational analysis between the VAS score of* de qi *intensity and the MASS index (*n* = 14). The VAS score of* de qi *intensity and the MASS index indicated a positive relationship (Spearman's *ρ* = 0.581, 95% CI = 0.0730 to 0.850).

As shown in Figure 2, the VAS score of* de qi* intensity was associated with the subtotal score of phase 2 in ASQ (Spearman's *ρ* = 0.654, 95% CI = 0.190 to 0.880). There was a positive correlation between the MASS index and subtotal scores of phase 2 in ASQ as well, though statistically insignificant (Spearman's *ρ* = 0.488, 95% CI = −0.0569 to 0.809). The VAS score of* de qi *intensity and the MASS index indicated a positive relationship (Spearman's *ρ* = 0.581, 95% CI = 0.0730 to 0.850) (Figure 3). The subtotal scores of phase 1 (Spearman's *ρ* = 0.256, 95% CI = −0.655 to 0.378) and phase 3 (Spearman's *ρ* = 0.384, 95% CI = −0.184 to 0.760) in ASQ were less likely associated with the VAS score.

### 3.3. Comparison of Outcomes in the Groups with Different Levels of Perception

**Figure 4:**
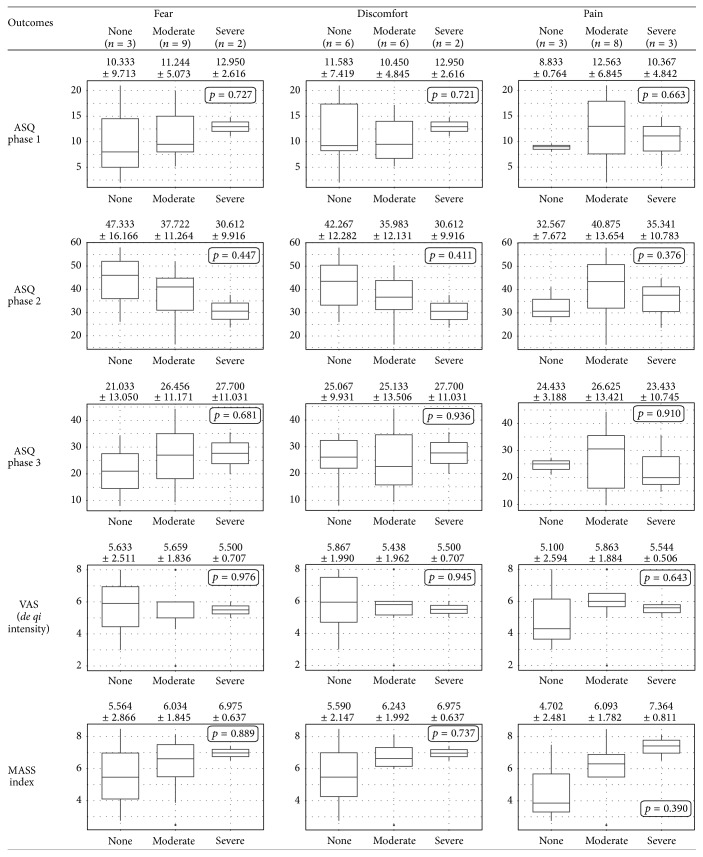
Comparisons of outcomes in the groups with different levels of unpleasant perception toward acupuncture treatment (*n* = 14). Boxes represent the interquartile range (IQR) between the first and the third quartiles. Lines inside the box indicate median. The upper/lower whiskers extend from the hinge to the highest/lowest value within 1.5 times the IQR. Data beyond this range were plotted as dots. Overall, unpleasant perception toward acupuncture did not correlate with* de qi *intensity, regardless of its type and degree.

Figure 4 shows the comparison of 5 outcomes (subtotal scores of ASQ phases 1–3, VAS score, and MASS index) in three different levels of “none,” “moderate,” and “severe,” according to each category (fear, discomfort, and pain). There was no significant difference in any of the outcomes between groups with different levels of perception. However, the MASS index mean showed an overall tendency to increase as the extent of fear, discomfort, and pain grew.

### 3.4. Correlational Analysis between the Individual Items of ASQ and MASS

**Figure 5:**
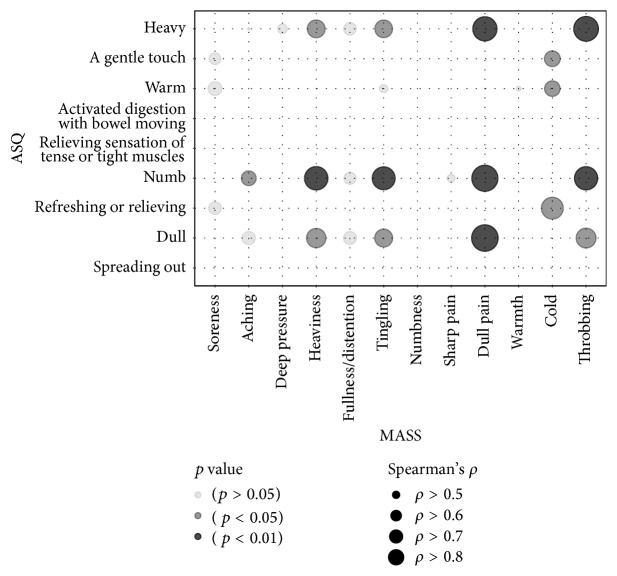
Correlational analysis between the items of Acupuncture Sensation Questionnaire (ASQ) and Massachusetts General Hospital Acupuncture Sensation Scale (MASS) (*n* = 14). Each circle represents a correlation between the categories from ASQ and MASS. The size of the circle reflects Spearman's rank correlation coefficients. Darker colors indicate lower *p* values. Correlations with Spearman's correlation coefficient over 0.4 were presented. MASS and ASQ showed strong correlations in certain analogous descriptors.

Results of Spearman's rank correlation test between the individual items of the ASQ and MASS are shown in Figure 5. Highly positive correlations were found between “dull” from the ASQ and “dull pain” from the MASS (Spearman's *ρ* = 0.847, *p* < 0.01), “numb” from the ASQ and “dull pain” from the MASS (Spearman's *ρ* = 0.833, *p* < 0.01), and “heavy” from the ASQ and “throbbing” from the MASS (Spearman's *ρ* = 0.789, *p* < 0.01). “Numb” and “dull” from the ASQ were the most significantly related items to MASS and were associated with five and four items from the MASS, respectively. “Heaviness,” “tingling,” “dull pain,” “cold,” and “throbbing” from the MASS were the most related items which showed highly positive and significant correlations with the ASQ items.

### 3.5. Subgroup Analysis between Male and Female Participants

To address potential gender-related discrepancies, we conducted subgroup analyses for the respective scores of each questionnaire. As shown in Table 4, the results for acupuncture perception questionnaire (fear (*p* = 0.365), discomfort (*p* = 0.416), and pain (*p* = 1)), ASQ (phase 1 (*p* = 0.240), phase 2 (*p* = 0.539), and phase 3 (*p* = 0.454)), VAS* de qi *intensity (*p* = 0.374), and MASS index (*p* = 0.733) were unaffected by gender variable.

## 4. Discussion

In this study, we first compared the* de qi* intensity score and MASS index with the subtotal scores at each phase of ASQ in a subset of healthy subjects who met the inclusion criteria. Initially devised by Kong et al., the MASS index is a single value that represents the full multivariate breadth and depth of acupuncture sensations. Through exponential smoothing, the index quantified the intensity of* de qi* regardless of the words used by the subject to describe the sensation, which in turn allowed for a more straightforward comparison [12]. The subtotal score of the manipulation stage in ASQ showed a direct correlation with the VAS* de qi* intensity score and MASS index. In other words, the general intensity of* de qi* was mostly derived from manipulation of the needle, rather than insertion or retention. This concurs with the previous results reported by Loyeung and Cobbin, in which the maintenance of needle sensation occurred only when the needle was both retained and manipulated [19]. A possible explanation for this relationship may be found in the tissue injury resulting from mechanical manipulation; this may lead to deformation of the extracellular matrix which elicits a signal transduction conveyed by various nerve fibers [20]. While such deformation is known to evoke “needle grasp” sensation felt by the practitioner, attempts to investigate it have been limited [21, 22]. With the introduction of a novel perception scale designed for practitioners, future researchers are encouraged to adopt and develop such scales so as to analyze the bilateral aspects of* de qi *[22].

Our study also found out that psychological factors such as the feeling of fear, discomfort, and pain toward acupuncture treatment did not affect the subjects' actual* de qi* sensations. In both questionnaires, the scores of acupuncture perception had poor correlations with the intensity of* de qi*. This is largely inconsistent with the previous report, which indicated that greater levels of anxiety and fear were related to heightened somatic focus [23]. Subjective cognitions such as expectation have been known to act as physiological modulators of perceptual and motor processes [24]. However, the descriptors used in this study reflected subjective psychological conditions rather than biological indexes and therefore may not correlate with such previous physiological studies. The participants in this study were homogenous in that they all had prior experience with acupuncture. In a previous study by Park et al., acupuncture-experienced subjects scored significantly lower for the intensity of certain sensations compared to naïve counterparts [15]. Taken together, one may assume that experience itself, rather than perception, affected the actual sensations perceived by the subject.

Although, in most cases, the expressions of acupuncture sensations differ between the two questionnaires, some uniform or analogous descriptors did exist, allowing for a correlational study among individual descriptors. In this study, we compared the scores of 12 predefined sensations in MASS with the sensations under the manipulation stage in ASQ. Interestingly, the “heaviness,” “tingling,” “dull pain,” and “throbbing” sensations of MASS were in high resemblance to one another in regard to the relevant descriptors of ASQ. Likewise, the “heavy,” “numb,” and “dull” sensations of ASQ showed a high resemblance to one another in regard to the relevant descriptors of MASS. Such resemblance of correlational patterns indicate that some descriptors of the same questionnaire were perceived as analogous, while the interfaces of such patterns suggest a matching point of view between the two questionnaires. Certain descriptors such as “spreading out” of the ASQ or “deep pressure” of MASS remained almost exclusive, without any relevance to the descriptors of the other questionnaire. The presence of independent dimensions allowed for the measurement of unique sensations that could only be identified by a specific questionnaire, which, in turn, provides the basis for simultaneous use of both in the assessment of* de qi* sensations. The acupoint (HT7) used in this study is located on the palmar wrist crease, where a rich network of tendon organs is observed. It has recently been hypothesized that manual acupuncture manipulation at such site mainly elicits numbness, heaviness, and distention [25]. Therefore, the dominant sensations reported in this study may be specific to such joint areas of the body. To further validate the relevance between ASQ and MASS, more research must be conducted in other acupoints of the cutaneous receptor or muscle spindle-rich areas.

This study had several limitations to consider. Above all, it was a single-center study with only 14 participants, in which the external validity was not fully guaranteed. Sample size calculation and a large-scale study are required for the proper generalization and confirmatory discussion. Future studies are also encouraged to implement a control group or comparison intervention with proper sample size calculation. Furthermore, the demographic data of participants showed an unequal distribution of gender, which led to an overrepresentation of female subjects. Females are reportedly associated with greater pain sensitivity than males, in part due to estrogen actions on nociceptive transmission [26, 27]. Although the results were unaffected by gender variables under subgroup analysis, a more balanced comprehensive sample is needed for validation of the results.

At present, there is no comprehensive method to assess the full dimensions of the* de qi* sensation. The comparison of two questionnaires with structural difference, namely, MASS and ASQ, demonstrates that a combined use of both may provide an extensive insight into the diverse aspects of* de qi*. The analysis of data obtained from this study builds on previous reports that intensity of* de qi* is mostly decided by the manipulation of the acupuncture needle, rather than insertion or retention [28, 29]. Likewise, further correlational studies may be conducted to elicit additional outcomes with good quality by the utilization of multiple* de qi *questionnaires in large-scale samples.

## Figures and Tables

**Table 1:** Perception toward acupuncture treatment, expressed as numbers (percentage) (*n* = 14).

	None	Moderate	Severe	Very severe
Fear	3 (21.4)	9 (64.3)	2 (14.3)	0 (0)
Discomfort	6 (42.9)	6 (42.9)	2 (14.3)	0 (0)
Pain	3 (21.4)	8 (57.1)	3 (21.4)	0 (0)

**Table 2:** * De qi* sensation measured with ASQ (*n* = 14).

Phase	Site of sensation	Sensations	Scores (95% CI)
Phase 1 (needle inserted)	Sensations at the needle site	Dull	4.16 (2.82, 5.51)
		Numb	4.00 (2.71, 5.29)
	Bodily sensation	Refreshing or relieving	3.13 (1.97, 4.29)

Phase 2 (needle manipulated)	Sensations at the needle site	Spreading out	4.97 (3.87, 6.08)
		Dull	5.73 (4.30, 7.16)
		Numb	5.52 (4.26, 6.78)
		Relieving sensation of tense or tight muscles	3.34 (2.09, 4.67)
		Warm	2.83 (1.83, 3.83)
		A gentle (soft) touch	3.48 (2.06, 4.90)
		Heavy	5.31 (3.67, 6.96)
	Bodily sensation	Refreshing or relieving	3.86 (2.63, 5.09)
		Activated digestion with bowel moving	2.86 (1.26, 4.45)

Phase 3 (needle retained)	Sensations at the needle site	Warm	2.29 (1.37, 3.21)
		Compressing or pressuring	3.68 (2.31, 5.04)
		Spreading out	4.43 (3.21, 5.65)
		Heavy	4.70 (3.29, 6.11)
	Bodily sensation	Refreshing or relieving	3.00 (1.80, 4.20)
		Surging open flow of stuffed or choked feeling	3.67 (2.15, 5.20)
		Activated blood circulation	3.71 (2.19, 5.22)

**Table 3:** VAS score of *de qi *intensity and *de qi* sensation measured with MASS and MASS index (*n* = 14).

	Scores (95% CI)
Intensity of *de qi *(VAS)	5.63 (4.62, 6.65)
Soreness	3.52 (2.31, 4.73)
Aching	4.30 (3.23, 5.37)
Deep pressure	4.84 (3.67, 6.02)
Heaviness	5.37 (4.08, 6.66)
Fullness/distention	4.37 (3.09, 5.65)
Tingling	4.52 (2.94, 6.10)
Numbness	3.11 (1.77, 4.45)
Sharp pain	2.44 (1.14, 3.75)
Dull pain	4.98 (3.98, 5.98)
Warmth	2.45 (1.29, 3.61)
Cold	2.15 (1.21, 3.09)
Throbbing	4.49 (3.24, 5.73)
Other	3.67 (2.22, 5.12)
MASS index	6.07 (4.98, 7.16)

**Table 4:** Gender differences in the scores of acupuncture perception questionnaire, ASQ, VAS *de qi *intensity, and MASS index (*n* = 14). The results for ASQ, VAS *de qi *intensity, and MASS index are expressed as mean ± SD. Overall, the results were unaffected by gender variable.

	Fear			Discomfort			Pain			ASQ phase 1	ASQ phase 2	ASQ phase 3	VAS (*de qi *intensity)	MASS index
	None (*n* = 3)	Moderate (*n* = 9)	Severe (*n* = 2)	None (*n* = 6)	Moderate (*n* = 6)	Severe (*n* = 2)	None (*n* = 3)	Moderate (*n* = 8)	Severe (*n* = 3)					
Male	0	4	0	1	3	0	1	2	1	8.425 ± 2.474	34.725 ± 6.757	21.850 ± 6.268	5.233 ± 0.746	6.323 ± 1.786
Female	3	5	2	5	3	2	2	6	2	12.440 ± 6.194	39.182 ± 13.488	26.920 ± 6.234	5.790 ± 2.043	5.965 ± 2.015
*p *value	0.365			0.416			1			0.240	0.539	0.454	0.374	0.733
